# Parental self-medication with antibiotics for children promotes antibiotic over-prescribing in clinical settings in China

**DOI:** 10.1186/s13756-020-00811-9

**Published:** 2020-09-07

**Authors:** Jiayao Xu, Xiaomin Wang, Kai Sing Sun, Leesa Lin, Xudong Zhou

**Affiliations:** 1 Institute of Social Medicine, School of Medicine, Zhejiang University, Hangzhou, China; 2 Department of Family Medicine and Primary Care, The University of Hong Kong, Hong Kong, China; 3 Department of Infectious Disease Epidemiology, London School of Hygiene and Tropical Medicine, London, UK

**Keywords:** Antibiotic use, Antibiotic prescription, Medical decision making, Children, Clinical

## Abstract

**Background:**

Self-medication with antibiotics (SMA) is one of the most dangerous inappropriate antibiotic use behaviors. This study aims to investigate the impact of parental SMA for children before a consultation on their doctor’s subsequent antibiotic prescribing behavior, including intravenous (IV) antibiotic use in the clinical setting of China.

**Methods:**

A cross-sectional survey was conducted between June 2017 and April 2018 in three provinces of China. A total of 9526 parents with children aged 0–13 years were investigated. Data from 1275 parents who had self-medicated their children and then visited a doctor in the past month were extracted and analyzed.

**Results:**

One-third (410) of the studied children had parental SMA before the consultation and 83.9% of them were subsequently prescribed antibiotics by doctors. Children with parental SMA were more likely to be prescribed antibiotics (aOR = 7.79, 95% CI [5.74–10.58]), including IV antibiotics (aOR = 3.05, 95% CI [2.27–4.11]), and both oral and IV antibiotics (aOR = 3.42, 95% CI [2.42–4.84]), than children without parental SMA. Parents with SMA behaviors were more likely to request antibiotics (aOR = 4.05, 95% CI [2.59–6.31]) including IV antibiotics (aOR = 2.58, 95% CI [1.40–4.76]), and be fulfilled by doctors (aOR = 3.22, 95% CI [1.20–8.63]).

**Conclusions:**

Tailored health education for parents is required in both community and clinical settings to discourage parental SMA for children. The doctors should not prescribe unnecessary antibiotics to reinforce parents’ SMA behaviors. We recommend expanding the current IV antibiotics ban in outpatient settings of China to cover outpatient pediatrics.

## Introduction

Antimicrobial resistance (AMR) is recognized as one of the biggest threats facing global health; inappropriate use of antibiotics, including antibiotic misuse and overuse in both community and clinical settings, is a major contributor to AMR [1, 2]. Self-medication with antibiotics (SMA) is one of the most dangerous and prevalent inappropriate antibiotic use behaviors, with a particularly high prevalence in low- and middle- income countries (LMICs) [3–5]. Despite this, antibiotics are the most commonly prescribed medicine for children [6], especially in LMICs where inappropriate antibiotic prescribing is prevalent [7].

A few studies have demonstrated the association between the SMA behavior before a consultation and doctors’ practices in clinical settings. A qualitative study conducted across nine European cities revealed that, when noticing their patients had started using antibiotics before the consultation, doctors would advise patients to complete their course of antibiotics, even if they thought antibiotics were unnecessary [8]. Another quantitative study conducted in a city in Poland indicated that doctors were more likely to prescribe antibiotics (aOR = 4.11) when patients had self-medicated with antibiotics before the consultation [9]. However, previous studies on this topic were conducted in only one country, where the prevalence of SMA and inappropriate antibiotic prescribing was relatively low, and the children were not specifically targeted. Our study aims to examine the impact of parental SMA for children on antibiotic prescribing in clinical settings in China, where both SMA and inappropriate antibiotic prescribing are prevalent.

In China, antibiotics have been pervasively used for children in both the community and clinical settings. In the community setting, it was reported that 59.4% of urban and 62% of rural parents had self-medicated their children with antibiotics in the past year [10, 11]. The antibiotics that parents used for self-medicating their children were mainly from over-the-counter purchases (35.3%), and also leftover antibiotics from previous prescriptions (63.1%) [12]. Although the Chinese government has officially banned non-prescription dispensing of antibiotics since 2004 [13], consumers in community and online pharmacies are still able to obtain antibiotics for self-medication without prescriptions [14, 15], which could be mainly due to fierce competition in the pharmacy market, consumers’ irrational expectations, the Food and Drug Administration’s limited capacity for supervision and minimal penalty for violating the regulation [16]. In the clinical setting, it was estimated that the average antibiotic prescription rate for children was 67.8% [17]. Moreover, among those who were prescribed antibiotics for upper respiratory infection (URI), 52.9% were given intravenous (IV) antibiotics [18]. Antibiotic over-prescribing is mainly blamed on doctors’ perverse economic incentives, lack of knowledge, and inadequate training [19–21]. However, the risk factors for SMA from the patient side have rarely been studied. Two previous studies found that patients with better antibiotic use knowledge were less likely to be prescribed antibiotics when seeing a doctor [22, 23]. However, few studies have investigated the impact of parental SMA for children before a consultation on their doctor’s subsequent antibiotic prescribing behavior.

Consequently, this study aims to investigate the impact of parental SMA for children before a consultation on antibiotic prescribing behaviors, including IV antibiotic use in China.

## Methods

### Study design and population

The reported data in this study came from a cross-sectional survey conducted between June 2017 and April 2018 in China. Parents with children aged 0–13 years were recruited across three purposefully selected Chinese provinces – Zhejiang, Shaanxi, and Guangxi – representing different geographic areas and varying economic development stages. Detailed methods, including sampling and recruitment, have been published elsewhere [12]. A representative sample of parents was obtained with a multistage stratified random cluster sampling procedure. The sampling procedure was conducted in four stages- provinces, prefecture-level cities, urban, and rural areas. The sampling sites included primary schools for parents whose children aged 6 to 13 years old, kindergartens for parents whose children aged 3 to 5 years old, and vaccination sites of community health centers for parents whose children aged 0 to 2 years old. The total sample size was 9526. Among them, 1275 parents had self-medicated their children and then visited a doctor in the past month.

### Measures

The items used in this study included three main parts: 1) the sociodemographic characteristics of parents and children- parents’ gender, education level, location of residence, medical education background, average household income, and their children’s gender and age, 2) parental self-medication behavior (with or without using antibiotics) for their children, and 3) children’s clinical consultation outcomes – whether children were prescribed antibiotics, route of antibiotics given if prescribed, if parents requested antibiotics, and, if requested, if their requests were fulfilled by doctors.

### Statistical analysis

Descriptive analyses were used to present weighted frequencies and percentages of factors of interest. Chi-square tests were conducted to examine the differences in hospital consultation experience between parents who self-medicated their children with antibiotics (SMA parents) and parents who self-medicated their children without using antibiotics (non-SMA parents). Multivariable logistic regression was adopted to examine the impact of parental SMA behavior for children on the outcome of their hospital consultation experience after controlling for socio-demographic factors. SPSS version 21.0 was used for statistical analyses. The significance level (type 1 error rate) was set at 0.05.

## Results

As is shown in Table 1, a total of 1275 children’s parents (451 from Guangxi, 438 from Shaanxi, 386 from Zhejiang) had self-medicated their children and then visited a medical institution in the preceding month. Half of the sampled children (51.6%) were boys, with an average age of 5.1 years (SD = 3.1), and 11.9% of them had parent with medical background. Most of the respondents were mothers (82.1%); 46.3% had a junior college and above education level; half of them had a monthly average household income over 5000 RMB (US$769), and 58.2% lived in urban areas.

**Table 1:** Sociodemographic characteristics of children and parents (*n* = 1275)

	N (%)
Province	
Guangxi	451 (35.4)
Shaanxi	438 (34.4)
Zhejiang	386 (30.3)
Gender of children	
Male	658 (51.6)
Female	617 (48.4)
Age of children (Mean, SD)	5.1 (3.1)
Relationship with children	
Father	228 (17.9)
Mother	1047 (82.1)
Education level	
Middle school and under	340 (26.7)
High school	345 (27.1)
Junior college and above	590 (46.3)
Residence	
Rural	533 (41.8)
Urban	742 (58.2)
Parent with medical background	
Yes	152 (11.9)
No	1123 (88.1)
Average monthly household income (RMB)	
< =3000 (US$461)	246 (19.3)
3001–5000 (US$462–769)	390 (30.6)
5001–10,000 (US$770–1538)	388 (30.4)
> =10,001 (US$1538)	251 (19.7)

Of all the children, 410 (32.2%) had been self-medicated by their parents with antibiotics before the consultation. Table 2 shows that a total of 693 (54.4%) children were prescribed antibiotics by doctors. Out of 1275 children, 448 (35.1%) were prescribed a route of oral antibiotics only, 76 (6.0%) with IV antibiotics only, and 169 (13.3%) with both oral and IV antibiotics. One hundred (7.8%) parents asked for antibiotics (4.2% for oral and 3.7% for IV antibiotic use) during a consultation and 71% of them had this request fulfilled.

**Table 2:** Chi-square tests for parental SMA and antibiotic prescribing in clinical settings (*n* = 1275)

	Self-medication before consultation		χ^2^	*p*
	Without antibiotics N (%)	With antibiotics N (%)		
Antibiotics prescribed by doctors			212.7	< 0.0001
No	516 (59.7)	66 (16.1)		
Yes	349 (40.3)	344 (83.9)		
Route of antibiotic prescribed			218.6	< 0.0001
No antibiotics prescribed	516 (59.7)	66 (16.1)		
Oral only	233 (26.9)	215 (52.4)		
Intravenous only	43 (5.0)	33 (8.0)		
Both oral and intravenous	73 (8.4)	96 (23.4)		
Asked for antibiotics			47.32	< 0.0001
No	828 (95.7)	347 (84.6)		
Yes	37 (4.3)	63 (15.4)		
Route of antibiotics parents asked for			49.71	< 0.0001
No requires	828 (95.7)	347 (84.6)		
Oral	16 (1.8)	37 (9.0)		
Intravenous	21 (2.4)	26 (6.3)		
Fulfilled parental request for antibiotics (*n* = 100)			8.191	0.004
Yes	20 (54.1)	51 (81.0)		
No	17 (45.9)	12 (19.0)		

Compared with children without parental SMA, those with parental SMA were more than two times likely to be prescribed antibiotics (83.9% vs. 40.3%, *p* < 0.0001). Children with parental SMA were prescribed higher percentages of oral, IV, and both oral and IV antibiotics than were those without parental SMA (52.4% vs. 26.9, 8.0% vs. 5.0, 23.4% vs. 8.4%, *p* < 0.0001). A higher proportion of SMA parents asked for antibiotics (15.4% vs. 4.3%, *p* < 0.0001), including oral antibiotics (9.0% vs. 1.8%, *p* < 0.0001) and IV antibiotics (6.3% vs. 2.4%, *p* < 0.0001) compared with non-SMA parents during consultations. A higher proportion of SMA parents’ requests for antibiotics were fulfilled by doctors than those of non-SMA parents (81.0% vs. 54.1%, *p* = 0.004).

After controlling for sociodemographic characteristics, our logistic regression (Table 3) indicates that children with parental SMA were more likely to be prescribed antibiotics (aOR = 7.79, 95% CI [5.74–10.58]), including IV antibiotics (aOR = 3.05, 95% CI [2.27–4.11]), and both oral and IV antibiotics (aOR = 3.42, 95% CI [2.42–4.84]), than children without parental SMA. SMA parents were more inclined to ask for antibiotics (aOR = 4.05, 95% CI [2.59–6.31]) and IV antibiotics (aOR = 2.58, 95% CI [1.40–4.76]), and their antibiotic requests were more likely to be fulfilled by doctors (aOR = 3.22, 95% CI [1.20–8.63]) than those of non-SMA parents.

**Table 3:** Logistic regression for the association between parental SMA and antibiotic prescribing in clinical settings

	Prescribed antibiotics by doctors (*n* = 1275)	Prescribed IV antibiotics by doctors ^#^ (n = 1275)	Prescribed both oral and IV antibiotics by doctors (n = 1275)	Asked for antibiotics (n = 1275)	Asked for IV antibiotics (n = 1275)	Antibiotic requests fulfilled by doctors (n = 100)
SMA for children						
No	Ref	Ref	Ref	Ref	Ref	Ref
Yes	7.79 (5.74,10.58)^***^	3.05 (2.27,4.11)^***^	3.42 (2.42,4.84)^***^	4.05 (2.59,6.31)^***^	2.58 (1.40,4.76)^**^	3.22 (1.20,8.63)^*^
Province						
Guangxi	Ref	Ref	Ref	Ref	Ref	Ref
Shaanxi	0.79 (0.58,1.07)	0.83 (0.58,1.17)	0.59 (0.39,0.88)^**^	0.89 (0.53,1.48)	0.69 (0.34,1.39)	2.44 (0.65,9.26)
Zhejiang	0.67 (0.48,0.96)^*^	1.37 (0.90,2.06)	0.92 (0.57,1.50)	1.34 (0.71,2.52)	0.56 (0.22,1.41)	2.05 (0.40,10.48)
Gender of children						
Male	Ref	Ref	Ref	Ref	Ref	Ref
Female	0.90 (0.71,1.16)	0.79 (0.59,1.05)	0.84 (0.60,1.18)	1.19 (0.78,1.81)	1.27 (0.70,2.32)	1.56 (0.54,4.53)
Age of children (Mean, SD)	1.01 (0.97,1.05)	1.01 (0.96,1.06)	1.02 (0.96,1.08)	1.04 (0.97,1.12)	1.08 (0.98,1.19)	0.94 (0.81,1.10)
Relationship with children						
Father	Ref	Ref	Ref	Ref	Ref	Ref
Mother	1.36 (0.99,1.88)	0.75 (0.52,1.07)	0.88 (0.57,1.35)	0.53 (0.33,0.87)^*^	0.65 (0.32,1.29)	1.94 (0.63,5.98)
Education level						
Middle school and under	Ref	Ref	Ref	Ref	Ref	Ref
High school	1.24 (0.87,1.77)	1.17 (0.78,1.74)	1.38 (0.87,2.18)	0.59 (0.34,1.04)	0.45 (0.20,1.00)^*^	0.59 (0.16,2.16)
Junior college and above	1.34 (0.93,1.94)	1.19 (0.78,1.81)	1.13 (0.69,1.86)	0.52 (0.28,0.95)^*^	0.40 (0.17,0.95)^*^	1.51 (0.36,6.30)
Residence						
Rural	Ref	Ref	Ref	Ref	Ref	Ref
Urban	1.36 (1.04,1.78)^*^	0.92 (0.67,1.25)	0.91 (0.63,1.31)	1.01 (0.63,1.60)	0.88 (0.45,1.69)	0.68 (0.20,2.35)
Parent with medical background						
Yes	Ref	Ref	Ref	Ref	Ref	Ref
No	1.45 (0.98,2.13)	1.63 (0.97,2.73)	1.67 (0.88,3.16)	0.92 (0.45,1.89)	1.54 (0.45,5.22)	1.89 (0.41,8.76)
Average monthly household income (RMB)						
< =3000	Ref	Ref	Ref	Ref	Ref	Ref
3001–5000	1.19 (0.82,1.74)	0.74 (0.49,1.12)	0.86 (0.53,1.38)	1.01 (0.56,1.82)	1.18 (0.51,2.74)	2.25 (0.55,9.17)
5001–10,000	1.11 (0.73,1.67)	0.71 (0.44,1.12)	0.81 (0.47,1.40)	0.84 (0.41,1.71)	1.93 (0.73,5.12)	1.50 (0.26,8.53)
> =10,001	1.04 (0.63,1.71)	0.52 (0.29,0.93)^*^	0.67 (0.34,1.33)	0.82 (0.34,1.95)	1.50 (0.42,5.38)	3.16 (0.39,25.90)

IV, intravenous

^#^Prescribed IV antibiotics including prescriptions contain IV antibiotics and both IV and oral antibiotics

^*^p < 0.05, ^**^p < 0.01, ^***^p < 0.001

Note: this table should appear on page 8 between line138 and line 139

## Discussion

To our knowledge, this is the first study to investigate the impact of parental SMA for children on doctors’ antibiotic prescribing in clinical settings in China. Our results demonstrated that children with parental SMA prior to a consultation were more likely to be prescribed antibiotics both oral and IV antibiotics during a clinical visit. Additionally, parents who have self-medicated their children before a consultation were more likely to ask for antibiotics, including IV antibiotics, during the consultation, and their requests were more likely to be fulfilled by doctors.

Consistent with the study in Poland [9], our study showed that SMA before consultation promoted inappropriate antibiotic prescribing in clinical settings. However, our study predicted an adjusted odds ratio in China (aOR = 7.79) which was a double of that in the Polish study (aOR = 4.11). This difference might due to our specific focus on children as the study population, and a higher prevalence of SMA in China. The huge difference in odds ratio between the Polish study and ours indicates that parental SMA for children before a consultation promotes doctors’ over-prescribing. This deserves further study especially in other LMICs, where both the prevalence of SMA and antibiotic prescription rate are high.

Our study found that SMA parents were more likely to ask for antibiotics and their requests were more likely to be fulfilled by the doctors, which explains how SMA triggered antibiotic prescriptions in clinical settings. Consistent with previous studies, parents who self-medicated their children with antibiotics were more likely to expect to receive antibiotics when seeing a doctor [24]. Given the currently tense doctor-patient relationship in China [25], defensive medicine (medical practice based on fear of legal liability rather than on patients’ best interests) [26] has worsened the situation [27]. It is difficult for doctors to refuse patients’ requests in China. A qualitative study suggested that doctors would choose to fulfill patients’ or their caregivers’ requests for antibiotics to avoid a quarrel, even antibiotics were unnecessary [28].

In China, IV antibiotics are frequently prescribed for self-limiting conditions [29]. In our study, 35.4% of those who had been prescribed antibiotics received IV antibiotics. This finding is consistent with previous studies that demonstrated IV antibiotics were frequently prescribed for outpatient pediatrics in China [18]. Our results showed higher prescription rates of IV antibiotics (alone or in combination with oral antibiotics) among children with parental SMA than those among children without parental SMA. However, most of our participants reported self-limiting symptoms, including cold (85.6%) and sore throat (53.9%), with some overlap between symptoms, for which antibiotics were unnecessary [30]. All of these children were self-medicated by their parents prior to a consultation, yet only 32.2% were given antibiotics. Therefore, differences in disease severity cannot fully explain the differences in antibiotic prescription rates. This phenomenon might partly be due to prevalent beliefs among parents [31] and doctors [32] in China that IV antibiotics are more effective than the oral ones. In addition, we found that requests of IV antibiotics from SMA parents during consultations could also contribute to the high demand of IV antibiotic prescriptions. To solve the problem of IV antibiotic overuse and misuse in clinical outpatient settings, several provinces and cities have been piloting a ban of IV antibiotics in secondary and above hospital outpatient settings since 2016, but the ban did not include outpatient pediatrics [33, 34]. We propose expanding this ban to include pediatrics if it is expanded to a nationwide ban in the future.

Our study has several implications for future policies and interventions. From the doctors’ perspective, the finding that parental SMA behaviors influenced doctors’ prescription decisions suggests that future education training on doctor-patient communication is needed to prevent doctors from being influenced by parental SMA behaviors before the consultation. In addition, considering that parental antibiotic requests trigger inappropriate prescriptions, we recommend developing better communication practices between doctors and patients and cultivating among doctors an understanding of parents’ antibiotic expectations. In our study, 71% of parents who requested antibiotics were satisfied by their doctors, but most of the reported symptoms or diseases were self-limiting conditions, for which antibiotics are not recommended [30]. Previous studies have shown that parents who expected antibiotics for their children but received an explanation and contingency plan without antibiotics were as satisfied as those whose expectation was met [35]. Moreover, a reduction in antibiotic prescriptions was resulted from a French antimicrobial stewardship program that encouraged a consultation with doctors to explain antibiotic safety and establish a trusting doctor-patient relationship [36]. A systematic review indicated that antibiotics were expected by parents because they believed that antibiotics were effective for treating their children ‘s illness, relieving their symptoms, reducing their likelihood of re-consultation, and preventing infection to other family members [37].

Thus, it is important to encourage doctors to understand the reasons for parents’ antibiotic requests and expectations and to improve doctor-patient communication through tailored antimicrobial stewardship programs. Though it is challenging for pediatricians to distinguish a feverish child from a self-limiting viral infection since s/he could have a serious bacterial infection [38]. Instead of refusing to prescribe antibiotics, it has been recommended that doctors withhold antimicrobial treatment for non-severe infections of children [39]. There is currently a great opportunity to address this problem, as the Chinese government has launched a zero-mark-up policy aiming to eliminate the economic incentives for over-prescribing [40]. However, China is currently facing a pediatrician shortage [41]; pediatrics has been reported as the most crowded department in many hospitals and pediatricians were under much higher pressure from patients (i.e. children’s parents) than were doctors in other departments [42]. Consequently, further studies are needed to determine possible ways to improve doctor-patient communications in China.

From the patients’ perspective, future interventions targeting the factors that trigger SMA and their requests or expectations would help reduce inappropriate antibiotic use in both community and clinical settings. SMA in LMICs is promoted by easy access to antibiotics from over-the-counter pharmacies. Unlike high-income countries, antibiotics in LMICs are easily obtained in community settings without a prescription [5, 43]. Additionally, SMA is promoted by lack of antibiotic use knowledge [44], previous recovery experiences [45], and keeping antibiotics at home [46]. Consequently, campaigns targeting patients and the general public in community settings that seek to eliminate these factors would contribute to lowering the rate of inappropriate antibiotic prescribing in clinical settings. Moreover, from the relationship between doctors and patients, previous prescriptions were proved to be one of the triggers of SMA [45]. Thus, the doctors should not prescribe unnecessary antibiotics to reinforce parents’ SMA behaviors.

Our study revealed that parental SMA behaviors influenced doctors’ prescription decisions. However, to our best knowledge, there are no clear clinical guidelines for doctors to intervene when interacting with patients who overuse or misuse antibiotics at home. Doctors are placed in a vulnerable and confused position when facing SMA patients/parents.

## Limitations

The findings of this study should be evaluated in light of its limitations. First, we did not ask about the frequency of being prescribed antibiotics during a consultation, thus the prescription rate and IV antibiotic prescription rate might be underestimated for children who might have been taken to more than one health facility by their parents. Second, we could only determine the number of prescriptions that contained both oral and IV antibiotics without knowing whether these were oral transmitted into IV, IV transmitted into oral, or oral and IV simultaneously. Third, our study relied on self-reporting, which could contain recall bias. However, in order to limit recall bias, we asked for children’s symptoms within the last month in our study, whereas previous studies have asked for symptoms within the last six months or even year [10, 11, 44, 45].

### Conclusions

Parental SMA behavior for their children before a clinical consultation was significantly associated with subsequent antibiotic prescriptions by doctors. Additionally, parents who had conducted SMA for children were more likely to ask for antibiotics, including IV antibiotics, during consultations. Tailored health education for parents is required in both community and clinical settings to discourage parental SMA for children. Future antimicrobial stewardship programs should target both the doctors and the parents by tailored health education and promoting doctor-patient communication. The doctors should not prescribe unnecessary antibiotics to reinforce parents’ SMA behaviors. We highly recommend expanding the ban on antibiotics in outpatient settings to cover outpatient pediatrics.

## Data Availability

The datasets used and/or analysed during the current study are available from the corresponding author on reasonable request.

## Abbreviations

AMR: Antimicrobial resistance
SMA: self-medication with antibiotics
LMICs: low- and middle- income countries
URI: upper respiratory infection
IV: intravenous
SMA parents: parents who self-medicated their children with antibiotics
non-SMA parents: parents who self-medicated their children without using antibiotics
SD: standard deviation
OR: odds ratio
aOR: adjusted odds ratio
